# A case of pseudo-Kaposi sarcoma with chronic limb-threatening ischemia

**DOI:** 10.1186/s40792-024-01933-7

**Published:** 2024-06-06

**Authors:** Yuya Tamaru, Shinsuke Kikuchi, Takayuki Uramoto, Kazuki Takahashi, Keisuke Kamada, Yuri Yoshida, Daiki Uchida, Takuya Nishio, Takeshi Yamao, Shunta Ishitoya, Mari Kishibe, Masashi Inaba, Toshihiko Hayashi, Akemi Ishida-Yamamoto, Nobuyoshi Azuma

**Affiliations:** 1 Department of Vascular Surgery, Asahikawa Medical University, Midorigaoka-Higashi 2-1-1-1, Asahikawa, 078-8510 Japan; 2 Department of Plastic and Reconstructive Surgery, Asahikawa Medical University, Midorigaoka-Higashi 2-1-1-1, Asahikawa, 078-8510 Japan; 3 Department of Radiology, Asahikawa Medical University, Midorigaoka-Higashi 2-1-1-1, Asahikawa, 078-8510 Japan; 4 Department of Dermatology, Asahikawa Medical University, Midorigaoka-Higashi 2-1-1-1, Asahikawa, 078-8510 Japan; 5 Department of Vascular Surgery, Moriyama Hospital, Miyamae 2-1-1-6, Asahikawa, 078-8392 Japan

**Keywords:** Pseudo-Kaposi sarcoma, Chronic limb-threatening ischemia, Dermatitis, Arteriovenous malformation

## Abstract

**Background:**

Pseudo-Kaposi sarcoma (PKS) is a rare vascular proliferative disease, caused by arteriovenous malformation (AVM) and chronic venous insufficiency. The lesions are characterized by purple or reddish-brownish papules, plaques, and nodules. Although benign, it is clinically similar to Kaposi's sarcoma (KS), a malignant disease, and must be differentiated by histopathological examination. We report a rare case of PKS with chronic limb-threatening ischemia (CLTI).

**Case presentation:**

An 83-year-old man with diabetes mellitus (DM) presented to a local dermatology department with a complaint of a right second toe ulcer and was, thereby, referred to our department due to arterial bleeding during skin biopsy to exclude malignant diseases. Although the pulsation of dorsalis pedis artery of the affected limb was palpable, the skin perfusion pressure was only 20 and 30 mmHg on the dorsum and planter surface, respectively, indicating severe ischemia of toe and forefoot. Ultrasonography and computed tomography revealed an AVM around the right second metatarsophalangeal joint and occlusion of the right dorsalis pedis artery in the middle, indicating CLTI in the background. Pathological findings of the skin biopsy found capillary blood vessel proliferation, hemosiderin deposition, and extravascular red blood cell leakage in the dermal layer, which could be found in KS. However, CD34 was normally stained in the vascular endothelium, and human herpesvirus-8 staining was negative, resulting in the pathological diagnosis of PKS, a proliferative vascular lesion associated with AVM. The ulcer was spontaneously epithelialized, but 2 years later the ulcer recurred and infection developed, necessitating treatment for abnormal blood flow. Transarterial embolization using *N*-butyl 2-cyanoacrylate for the AVM controlled abnormal perfusion once; however, the procedure exacerbated perfusion of the toe, resulting in foot ulcer progression. Forefoot amputation with surgical excision of AVM was performed, and thereby, wound healing was achieved.

**Conclusion:**

This is a rare case of PKS with CLTI complicated with AVM. As there is currently no established consensus on the treatment of PKS, the approach to treatment strategy should be tailored to the specific condition of each patient.

## Background

Foot ulcers are associated with several factors, such as abnormal foot mechanics and peripheral neuropathies, including diabetic foot, peripheral artery disease, and chronic venous insufficiency. Dermatitis can cause skin inflammation, ulcer formation, and skin itchiness of the lower extremities. Vascular proliferative disorders also cause dermatitis, which can develop into foot ulcers due to increased vasculature in the cutaneous layer [1]. Both benign and malignant pathologies can contribute to vascular proliferative disorders. Therefore, pathological evaluation of skin biopsies is important for determining treatment strategies. Pseudo-Kaposi sarcoma (PKS), also known as acroangiodermatitis, is a rare proliferative vascular skin disease. Its pathology involves vascular malformations caused by arteriovenous malformations (AVMs) and chronic venous insufficiency. Clinically, skin lesions are characterized by purple or reddish-brownish papules, plaques, and nodules [2]. Although PKS is a benign disorder, it shares clinical similarities with Kaposi sarcoma (KS), a malignant skin disease caused by human herpes virus-8 (HHV-8) in people with human immunodeficiency virus (HIV). Both KS and PKS are vascular proliferation disorders of the skin and require differentiation through histopathological examination. PKS is not usually associated with limb ischemia. However, in older individuals with comorbid diabetes mellitus (DM), pathology and treatment can become more complex. The management of abnormal blood flow to the foot and preservation of foot arterial perfusion are essential in treating ischemic limb. In this report, we present a rare case of PKS with chronic limb-threatening ischemia (CLTI) caused by AVM and DM in a patient of advanced age.

## Case

**Fig. 1:**
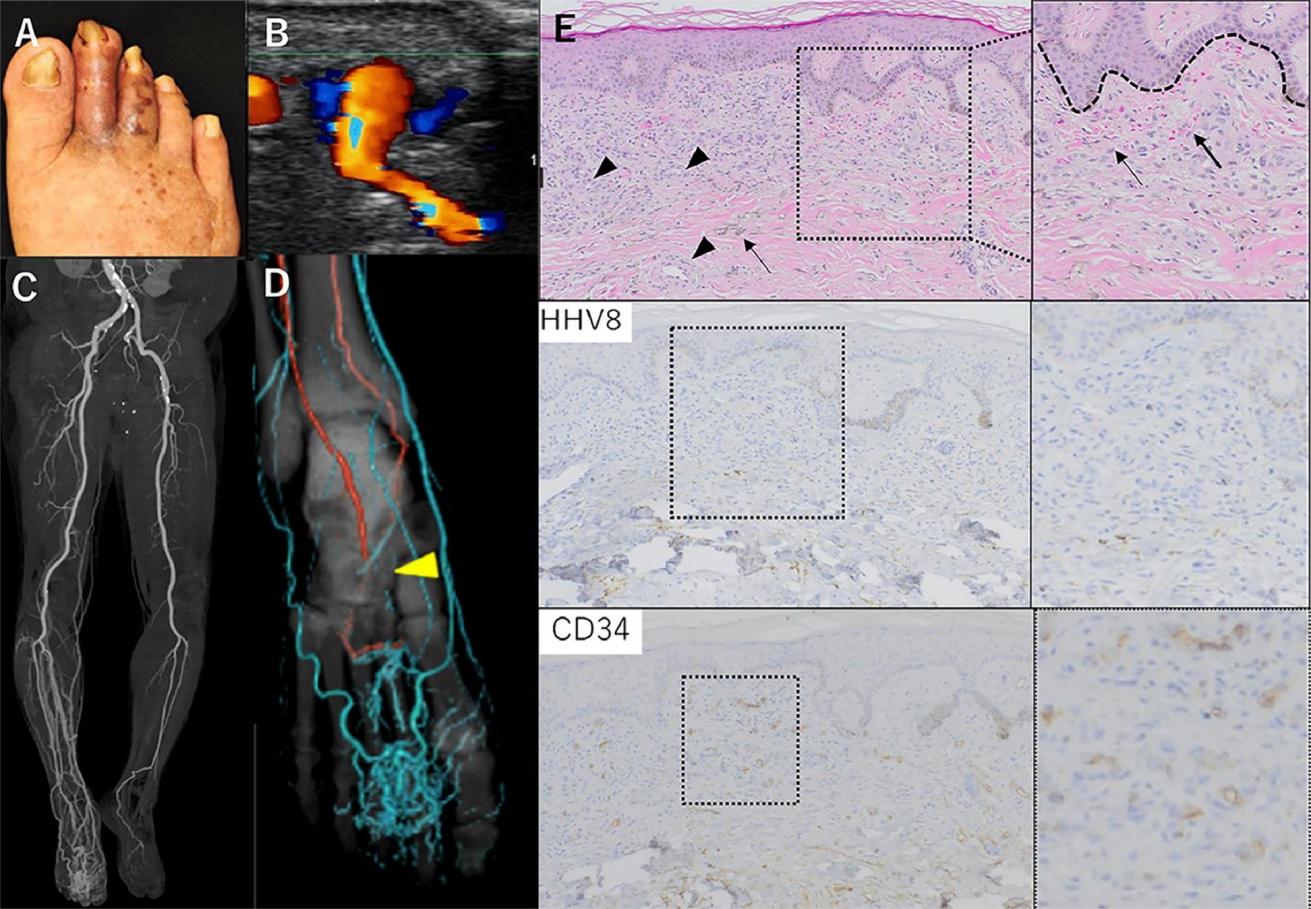
Preoperative findings. Color changes in the toes (**A**). Arteriovenous malformation (AVM) around the right third metatarsophalangeal joint detected by ultrasound sonography (**B**). Computed tomography angiography showed arterial and venous perfusion in the right lower extremity at the arterial phase and atherosclerotic lesions of the left infrapopliteal arteries (**C**). AVM around the right second metatarsophalangeal joint and atherosclerotic lesions in the right dorsalis pedis artery, indicated by arrowhead (**D**). Skin biopsy revealed dermal capillary proliferation by arrowhead, extravascular leakage of red blood cells by arrows, and hemosiderin deposition in the interstitium (H&E × 100). Human herpes virus-8 staining was negative (×100). CD34 staining was only positive in the vascular endothelial cells (×100) (**E**)

**Fig. 2:**
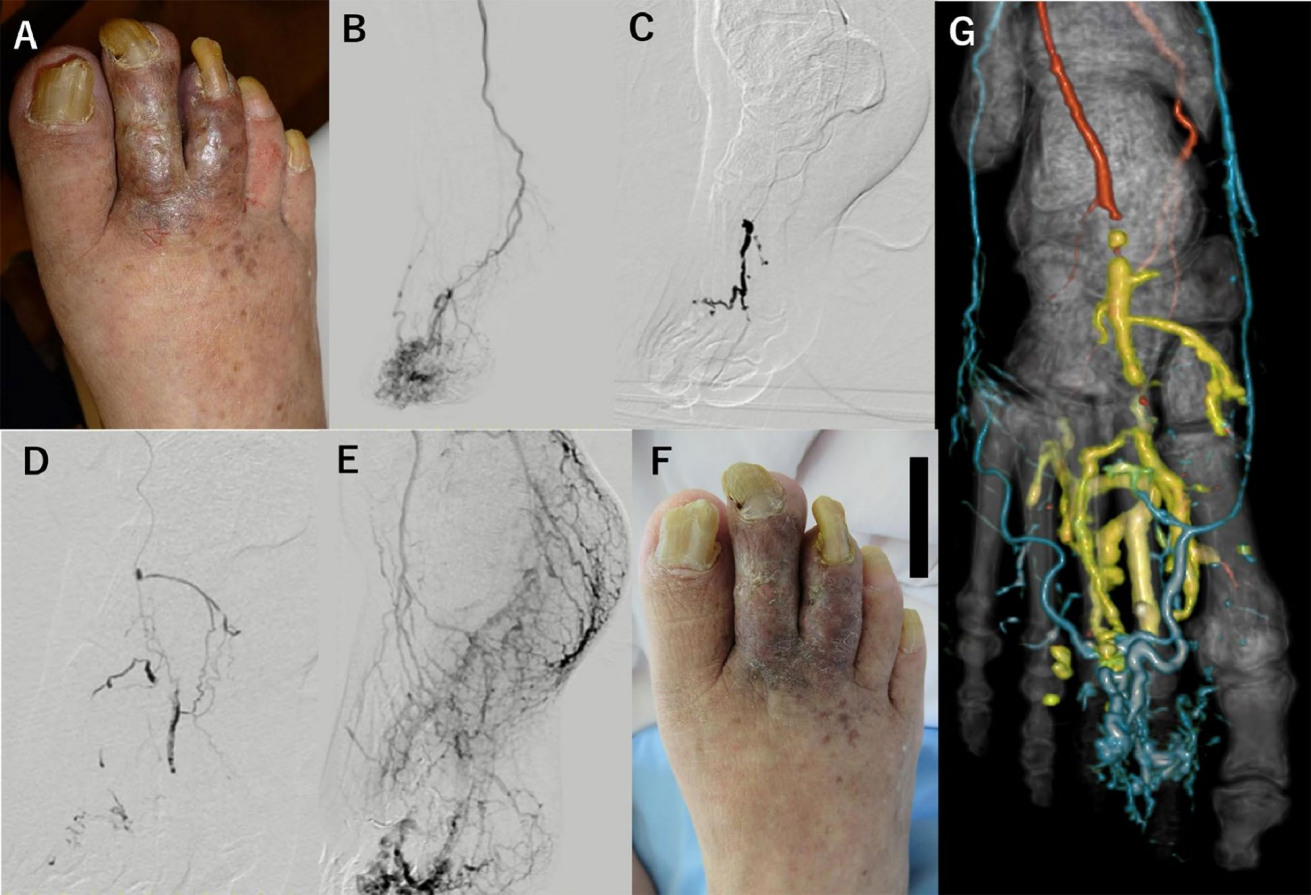
Right toe appearance and embolization findings. The second and third toes were infected (**A**). Digital subtraction angiography showed arteriovenous malformation (AVM) (**B**). Embolization using a mixture of *N*-butyl cyanoacrylate (NBCA) and lipiodol from the right plantar artery (**C**) and dorsalis pedis artery (**D**). Significant decrease in AVM perfusion after embolization (**E**). Infection of the toes was improved by antibiotic use and embolization (**F**). Computed tomography angiography after embolization showed decreased AVM flow compared to Fig. 1D. The embolic agent is indicated by the yellow segments (**G**)

**Fig. 3:**
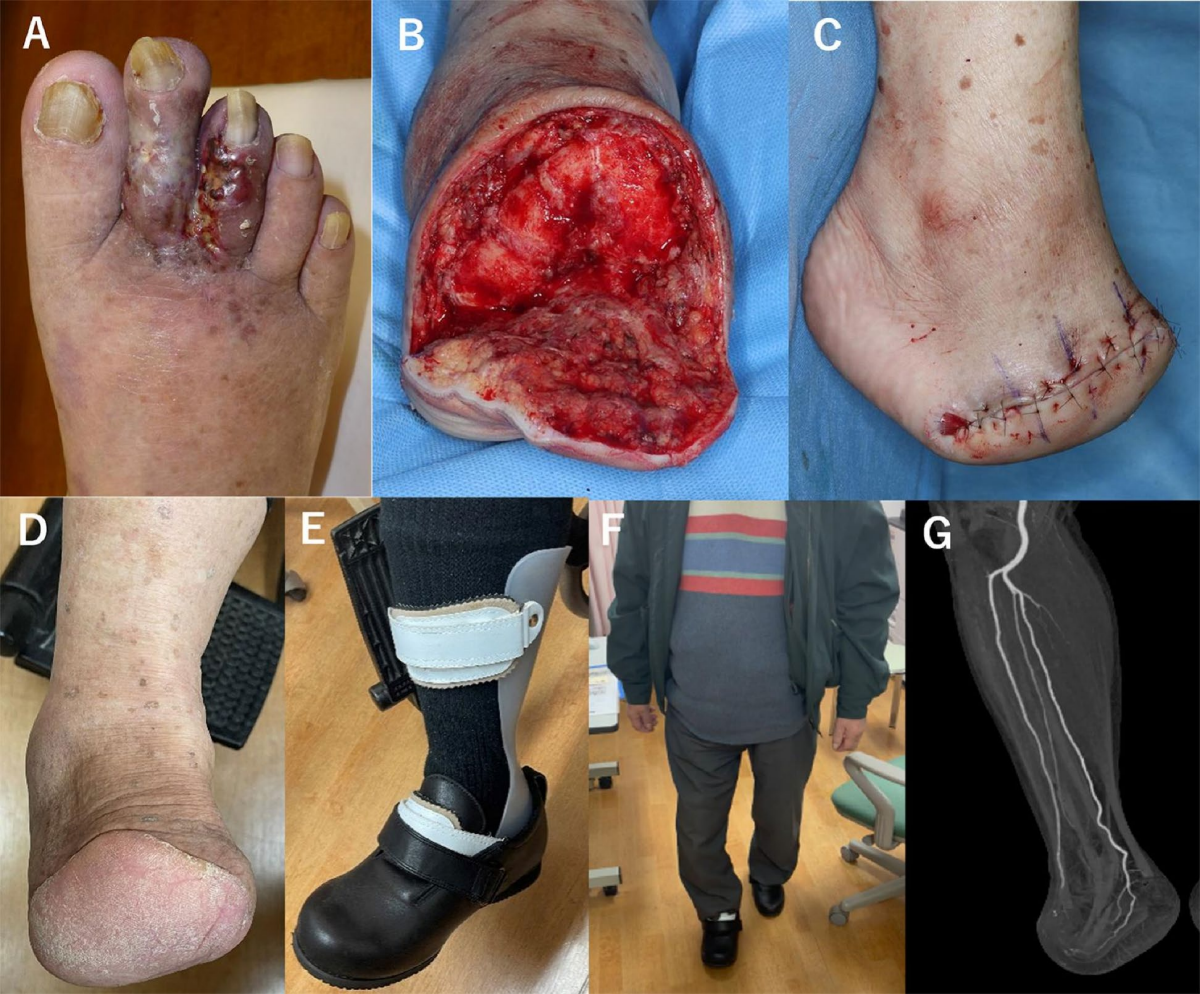
Ulceration and surgical resection of arteriovenous malformation (AVM). Ulcers occurred on the treated right toes after embolization (**A**). Disarticulation at the Lisfranc joint, including surgical resection of AVM (**B**, **C**). Wound healing and ambulation were achieved with assistance equipment (**D**–**F**). Postoperative computed tomography angiography showed that AVM was completely resected (**G**)

An 83-year-old man with DM and chronic kidney disease noticed color changes in his toes for 4 years and had small ulcers and purplish-reddish plaques on his right toe for 2 years. The patient received conservative treatment for the skin lesions at a local dermatology clinic, resulting in repeated exacerbations and remissions. Skin biopsy was performed to exclude malignant skin diseases. During that time, arterial bleeding from the punctured site was confirmed. Consequently, he was referred to our department with a possible AVM. During physical examination at his first visit to our department, dark reddish-brownish macules and ulcers were observed on the right second and third toes (Fig. 1A). Although the pulsation of the dorsalis pedis artery (DPA) of the affected limb was palpable and the ankle–brachial index was 1.10, the skin perfusion pressure was only 20 and 30 mmHg on the dorsal and plantar surface, respectively, indicating severe ischemia of toe and forefoot. Ultrasonography revealed an AVM around the right second metatarsophalangeal joint (Fig. 1B), which attributed to arterial steal phenomenon. Computed tomography (CT) angiography revealed an AVM around the right second metatarsophalangeal joint (Fig. 1C). The right DPA was occluded in the middle, raising suspicion of CLTI (Fig. 1D). Routine hematological and biochemical tests indicated elevated blood urea nitrogen and creatinine (28 and 1.7 mg/dl, respectively), and HbA1c was 7.1%. Serological testing for HIV yielded negative results. Skin biopsy revealed dermal capillary proliferation, extravascular leakage of red blood cells, and hemosiderin deposition in the interstitium. Immunohistochemical analysis revealed negative staining for HHV-8 and positive staining for CD34 in the vascular endothelial cells (Fig. 1E). Based on these results, a diagnosis of PKS with CLTI. The following points were discussed for treatment: (1) ligation of AVM was difficult due to bleeding from AVM; (2) embolization could control the AVM itself, however, ischemia in the toes, where blood flow is reduced by diabetic atherosclerosis, was thought to be exacerbated by embolization. Due to the difficulty of eliminating AVM without resorting to minor amputation, conservative treatment was recommended. The skin lesion was managed without ulcer recurrence for 2 years since it healed with conservative treatment, such as antibiotic ointments and Fiblast Spray, known as recombinant human basic fibroblast growth factor (KAKEN PHARMACEUTICAL CO., LTD. Tokyo, Japan). Unfortunately, the lesion finally deteriorated due to infection, after 2 years of follow-up (Fig. 2A), leading to increased foot pain, and C-reactive protein was detected (3.0 mg/dl). The patient was treated with intravenous ampicillin/sulbactam (1.5 g q12hr) for 14 days based on the wound culture results. Next, to manage this deterioration of the skin lesions, minor amputation was necessary, but arterial bleeding was identified during biopsy, so it was considered that amputation was high risk of bleeding. Therefore, endovascular embolization was primarily indicated because of controlling AVF flow for a preoperative adjuvant before minor amputation and reduction of the risk of bleeding during surgical resection, with risk of progression of foot ischemia. As a result, the patient was admitted and AVM was controlled through transarterial embolization. To perform embolization, a 4-French sheath was anterogradely inserted into the right common femoral artery. The nidus structure of the AVM was identified and embolized using a mixture of *N*-butyl cyanoacrylate (NBCA) and lipiodol at a 1:5 dilution, introduced through the DPA and plantar artery (Fig. 2B–E). The patient was discharged once the ulcer improved after the antibiotic treatment and embolization (Fig. 2F). This procedure resulted in decreased contrast enhancement of the nidus. CT angiography after embolization showed decreased AVM flow compared with before (Fig. 2G). Unfortunately, he was readmitted owing to deterioration of the skin lesions on the toes 14 days after embolization (Fig. 3A). It could be deteriorated owing to progression of toe ischemia and infection caused by the embolizing to blood flow not only AVM but outside the AVM. To address this worsening situation, forefoot amputation was performed at the Lisfranc joint, accompanied by surgical resection of the AVM (Fig. 3B–C). The wound healed successfully, and gait function was regained with the use of assistance equipment (Fig. 3D–F). The entire treatment process required 4 months. Postoperatively, the patient was followed by CT angiography (Fig. 3G). No recurrence was observed for 1 year.

## Discussion

PKS is an angioproliferative dermatitis with clinical features similar to Kaposi sarcoma [3]. The pathogenesis of PKS is attributed to abnormal vascular perfusion, including chronic venous insufficiency and AVM. PKS can be classified into two pathological types: Mali type and Stewart–Bluefarb type. The Mali type is characterized by a low flow owing to venous insufficiency and is most commonly observed at early ages. In contrast, the Stewart–Bluefarb type involves high flow caused by AVM and is rare. Clinically, the Stewart–Bluefarb type resembles KS [4]. Development of PKS has also been associated with amputation stumps, paralyzed limbs, and AV shunts for dialysis [5, 6]. The cause of PKS is not generally understood. However, in this case, the diabetic foot might be associated with the development of PKS by skin microvascular dysfunction through the impairment of the complex interaction of neurogenic and neurovascular control [7]. Although the pathology underlying skin disorders triggered by abnormal vascular flow is not well understood, several hypotheses have been proposed: (1) the angiogenic response to high perfusion rates and elevated oxygen saturation can contribute to fibroblast proliferation and reactive endothelial hyperplasia [8, 9]. (2) Increased venous pressure resulting from AVM can stimulate endothelial proliferation [10]. (3) AVM accompanied by distal ischemia can cause local secretion of vascular endothelial growth factor, which plays a crucial role in vascular endothelial proliferation [11].

PKS can be differentiated from KS based on histopathological characteristics. Histologically, PKS exhibits capillary proliferation in the dermal layer, hemosiderin deposition in the dermis, extravasated erythrocytes, inflammatory mononuclear cell infiltration (including lymphocytes, histiocytes, eosinophils, and occasional plasma cells), and perivascular dermal fibroblasts. Histopathological differentiation between PKS and KS can be achieved through the expression of CD34 antigen and HHV-8. In KS, CD34 positivity is observed in endothelial and perivascular spindle cells, whereas perivascular cells in PKS completely lack CD34 expression. In addition, HHV-8 is exclusively present in KS [3].

For patients with AVM, surgical resection and embolization are required. Although not all cases of PKS exhibit distal ischemia, severe ischemia resulting from DM-related atherosclerosis played a significant role in the pathophysiology of the present case. The Stewart–Bluefarb type of PKS with CLTI, as observed in this particular case, is exceedingly rare, and this report represents the first documented case in the literature to the best of our knowledge. Although no consensus on treatment has been established, the fundamental treatment strategy involves conservative therapy, including compression bandages, rest, and limb elevation, and symptomatic treatment, including topical steroids and oral antibacterial agents [10, 12]. For the Stewart–Bluefarb type of PKS, the curative treatment involves the removal of the underlying vascular lesion through embolization or surgery. However, this can be challenging when peripheral arterial lesions are present [3, 13]. Embolization carries risks such as ulceration, necrosis, and infection, so the indications for embolization should be carefully evaluated [10, 14]. In the present case, the lesion was complicated by severe ischemia due to DM and AVM, with the risk of ischemic progression following embolization. Nevertheless, embolization was performed as the initial choice to control AVM and salvage the affected toes. Selective embolization can be achieved using particles such as gelfoam, ivalon, acrylates, absolute, and alcohol [13–15]. As the lesion located peripheral, NBCA was used as the embolization material to reach more peripheral areas. However, embolization led to ulceration and exacerbation of the infection, necessitating surgical resection. In a retrospective analysis of this case, it was observed that blood flow to the nidus structure primarily occurred through the plantar artery. Consequently, embolization of only the plantar artery may have been a better choice. Considering the low skin SSP value, surgical resection might have been a viable treatment option. Alternatively, embolization could be two-staged for each dorsalis and plantar area. If the ischemia was in a treatable site, revascularization could have been a useful treatment. In addition, surgery such as primary transmetatarsal amputation might have been a better first choice owing to the high risk of ulceration and infection following embolization caused by CLTI complications.

## Conclusion

Herein, we present a rare case of PKS with CLTI and AVM. As there is currently no established consensus on the treatment of PKS, the approach to treatment strategy should be tailored to the specific condition such as ischemia of distal tissue.

## Data Availability

The data used for this case report are available from the corresponding author upon request.

## Abbreviations

PKS: Pseudo-Kaposi sarcoma
AVM: Arteriovenous malformation
CLTI: Chronic limb-threatening ischemia
DM: Diabetes mellitus
KS: Kaposi sarcoma
HHV-8: Human herpes virus-8 (HHV-8)
HIV: Human immunodeficiency virus (HIV)
DPA: Dorsalis pedis artery
CT: Computed tomography
NBCA: *N*-Butyl cyanoacrylate
